# HHV-6 encephalitis in a non-transplanted adult acute myeloid leukemia patient

**DOI:** 10.1007/s00277-021-04409-y

**Published:** 2021-01-14

**Authors:** Margarete Voigt, Konrad Sinn, Amer Malouhi, Thomas Gecks, Jan Zinke, Inken Hilgendorf, Sebastian Scholl, Andreas Hochhaus, Ulf Schnetzke

**Affiliations:** 1 Klinik für Innere Medizin III, Fachbereich Endokrinologie, Universitätsklinikum Jena, Jena, Germany; 2 Klinik für Innere Medizin II, Abteilung für Hämatologie und Internistische Onkologie, Universitätsklinikum Jena, Am Klinikum 1, 07747 Jena, Germany; 3 Institut für Diagnostische und Interventionelle Radiologie, Universitätsklinikum Jena, Jena, Germany; 4 Klinik für Innere Medizin I, Internistische Intensivmedizin, Universitätsklinikum Jena, Jena, Germany; 5 Klinik für Neurologie, Klinikum St. Georg Leipzig, Leipzig, Germany

Dear Editor,

Here, we report a case of a clinically manifest human herpesvirus-6 (HHV-6) encephalitis in a neutropenic patient with acute myeloid leukemia (AML) in a non-transplant setting while on antimicrobial prophylaxis including aciclovir.

A 38-year-old female patient was admitted to the hospital with newly diagnosed AML. Following second consolidation chemotherapy containing high-dose cytarabin, the patient showed signs of forgetfulness, myoclonia in both arms, and eventually suffered from a generalized tonic-clonic seizure. Electroencephalography showed generalized slowing but no epileptiform activity. The MRI showed restricted diffusion bilateral within the frontal striatum, as well as increased contrast enhancement in the lenticulostriate arteries, indicative of inflammatory lesions (Fig. 1a). Polymerase chain reaction (PCR) analyses of the cerebrospinal fluid (CSF) and blood serum were positive for HHV-6. Intravenous antiviral therapy with ganciclovir was commenced. The patient continued to display amnesia of short-term memory, which was slowly declining within the following weeks. Repetitive MRI scans showed increased inflammatory changes 9 days later (Fig. 1b) that receded within the following 2 weeks with only residual inflammation within the basal ganglia. Twenty-five days following initiation of ganciclovir PCR for HHV-6 from blood serum was negative, and secondary prophylaxis with oral valganciclovir was started.

**Fig. 1:**
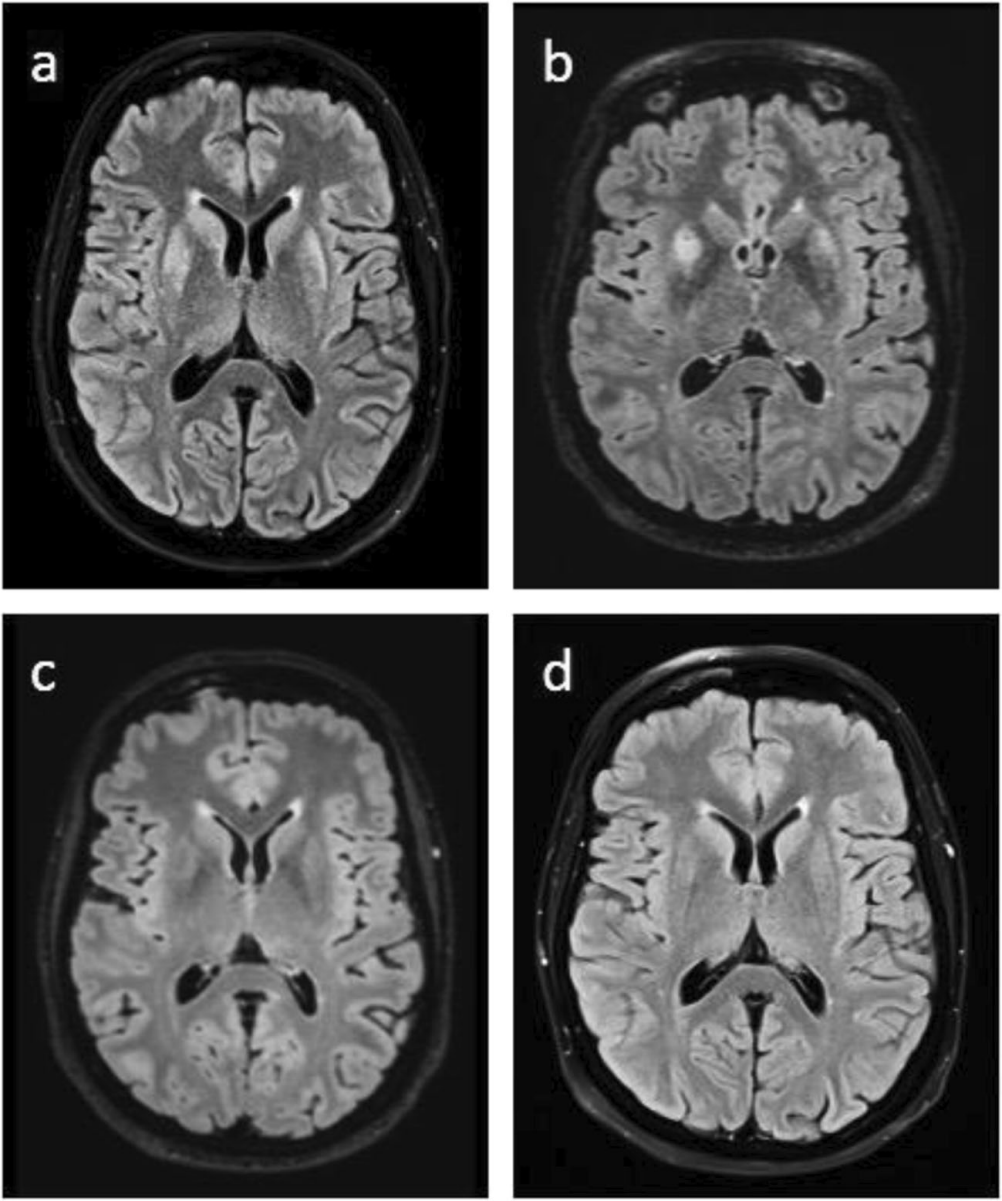
MRI T2-FLAIR imaging studies during the course of HHV-6 encephalitis. **a** Slight bilateral asymmetrical diffusion restricted areas in corpora striata. **b** Marked bilateral asymmetrical diffusion restricted areas in corpora striata. **c** Regression of diffusion restricted areas in corpora striata. **d** Normal appearance of corpora striata without evidence of diffusion restriction

With regard to the AML therapy, indication for allogeneic hematopoietic stem cell transplantation (allo-HCST) from an HLA-matched sibling was met due to ongoing detection of NPM1. Before allo-HSCT PCR for HHV-6 in CSF as well as blood was negative, and MRI scan showed few residual inflammatory signs within the basal ganglia (Fig. 1c). Allo-HSCT was performed after conditioning chemotherapy with treosulfan and fludarabine, and immunosuppression with cyclosporine and methotrexate was administered as described [1]. Importantly, no signs and symptoms of virus reactivation during and following allo-HSCT were noticed as the patient continued on prophylactic valganciclovir, continuously. An MRI scan 3 months after initial diagnosis of HHV-6 encephalitis and performed allo-HSCT displayed no signs of cerebral inflammation (Fig. 1d). Sixty days after allo-HSCT bone marrow biopsy showed a complete remission. Table 1 shows a time course of the AML therapy, MRD levels, HHV-6-PCR, and MRI scans.

**Table 1:** Time course including AML therapy, MRD, microbiologic tests, and MRI scans

Therapy	Induction therapy (7 + 3 protocol)	1st consolidation (HD-AraC)	2nd consolidation (HD-AraC)	Allo-HSCT	+ 31 days post allo-HSCT	+ 63 days post aloo-HSCT
Start date	17.01.2020	04.03.2020	12.05.2020	18.08.2020	17.09.2020	19.10.2020
NPM1 mutation (%)	2240	0.6	0.5	0.07	0.03	0.003
HHV-6-PCR-blood (days post chemotherapy			Positive (+ 34, + 50, + 66)	Negative		
HHV-6-PCR-CSF (days post chemotherapy)			Positive (+ 34)	Negative		
Chimerism (5)					89	100
MRI imaging (days post chemotherapy)			(Fig. 1a) + 34 (Figure 1b) +42	(Fig. 1c) − 8		(Fig. 1d) + 63

HHV-6 displays latency after the primary infection. End-organ disease usually occurs in an immunocompromised host and is most likely due to reactivation. HHV-6 is the most frequent cause of encephalitis in an allo-HSCT setting and typically takes a subacute course with slow beginning of confusion, amnesia, and change of personality [2]. Post allo-HSCT HHV-6 reactivation is associated with higher incidences of acute graft-versus-host disease, CMV reactivation, and non-relapse mortality [3]. Outcome of HHV-6 encephalitis post allo-HSCT is poor with a mortality rate of 20 to 40% and a high proportion of patients suffering from neurological sequelae including temporal lobe epilepsy [4, 5]. Treatment options include ganciclovir, foscarnet, and cidofovir that inhibit HHV-6 replication [6]. As HHV-6-reactivation often coincides with the onset of disease, routine screening is not recommended [7]. In 2019 guidelines for management of HHV-6 infection in patients with hematologic malignancies were published [8].

To our knowledge, this is the first report of clinical manifest HHV-6 encephalitis in a leukemic patient prior to allo-HSCT without any predisposing factors contributing to an immunocompromised state before the diagnosis of AML was made. Since MRI scan may be negative at the beginning of the disease, close clinical and diagnostic monitoring is necessary if a patient presents with acute-onset altered mental status (encephalopathy), short-term memory loss, or seizures. This case indicates the significance of observing clinical signs and symptoms of incipient encephalopathy and rapid diagnostic and therapeutic measures to prevent residual damage.

## References

[1] Casper J, Holowiecki J, Trenschel R, Wandt H, Schaefer-Eckart K, Ruutu T, Volin L, Einsele H, Stuhler G, Uharek L, Blau I, Bornhaeuser M, Zander AR, Larsson K, Markiewicz M, Giebel S, Kruzel T, Mylius HA, Baumgart J, Pichlmeier U, Freund M, Beelen DW (2012). Allogeneic hematopoietic SCT in patients with AML following treosulfan/fludarabine conditioning. Bone Marrow Transplant.

[2] El-Jawahri AR, Schaefer PW, El Khoury JB, Martinez-Lage M (2018). Case 5-2018: a 63-year-old man with confusion after stem-cell transplantation. N Engl J Med.

[3] Aoki J, Numata A, Yamamoto E, Fujii E, Tanaka M, Kanamori H (2015). Impact of human herpesvirus-6 reactivation on outcomes of allogeneic hematopoietic stem cell transplantation. Biol Blood Marrow Transplant.

[4] Zerr DM (2006). Human herpesvirus 6 and central nervous system disease in hematopoietic cell transplantation. J Clin Virol.

[5] Ogata M, Oshima K, Ikebe T, Takano K, Kanamori H, Kondo T, Ueda Y, Mori T, Hashimoto H, Ogawa H (2017). Clinical characteristics and outcome of human herpesvirus-6 encephalitis after allogeneic hematopoietic stem cell transplantation. Bone Marrow Transplant.

[6] Prichard MN, Whitley RJ (2014). The development of new therapies for human herpesvirus 6. Curr Opin Virol.

[7] Ogata M, Satou T, J-i K, Saito N, Yoshida T, Okumura H, Ueki T, Nagafuji K, Kako S, Uoshima N (2013). Human herpesvirus 6 (HHV-6) reactivation and HHV-6 encephalitis after allogeneic hematopoietic cell transplantation: a multicenter, prospective study. Clin Infect Dis.

[8] Ward KN, Hill JA, Hubacek P, de la Camara R, Crocchiolo R, Einsele H, Navarro D, Robin C, Cordonnier C, Ljungman P (2019). Guidelines from the 2017 European Conference on Infections in Leukaemia for management of HHV-6 infection in patients with hematologic malignancies and after hematopoietic stem cell transplantation. Haematologica.

